# Retrocardiac Lucency in Neonates: Air Trapped in the Pulmonary Ligament

**DOI:** 10.5334/jbsr.2338

**Published:** 2021-01-29

**Authors:** Antoine Collignon, Dana Dumitriu

**Affiliations:** 1 Cliniques universitaires Saint Luc, BE

**Keywords:** chest, x-ray, neonate, pulmonary ligament, lucency, retrocardiac

## Abstract

Air trapped in neonates’ pulmonary ligament is often the consequence of positive pressure ventilation and its typical radiographic appearance must be recognized in order to prevent the use of aggravating factors.

**TEACHING POINT:** Air trapped in neonates’ pulmonary ligament is often the consequence of positive pressure ventilation; its typical waterdrop appearance must be recognized on radiographs to prevent unnecessary additional measures.

## CASE

A premature newborn was delivered by emergency C-section at 33 weeks of gestation, for fetal distress and intrauterine growth restriction. The baby received nasal continuous positive airway pressure (CPAP) ventilation immediately after birth.

**Figure 1:**
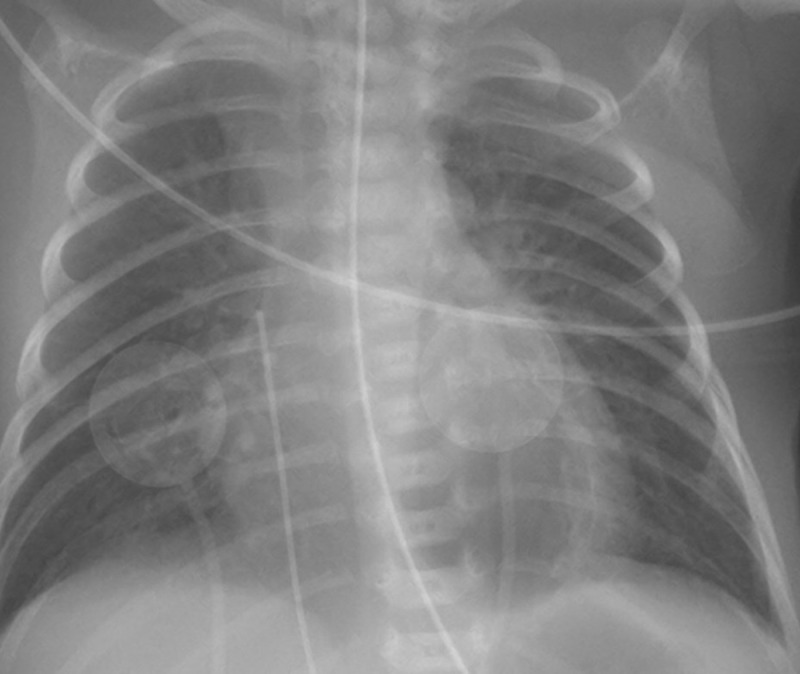


**Figure 2:**
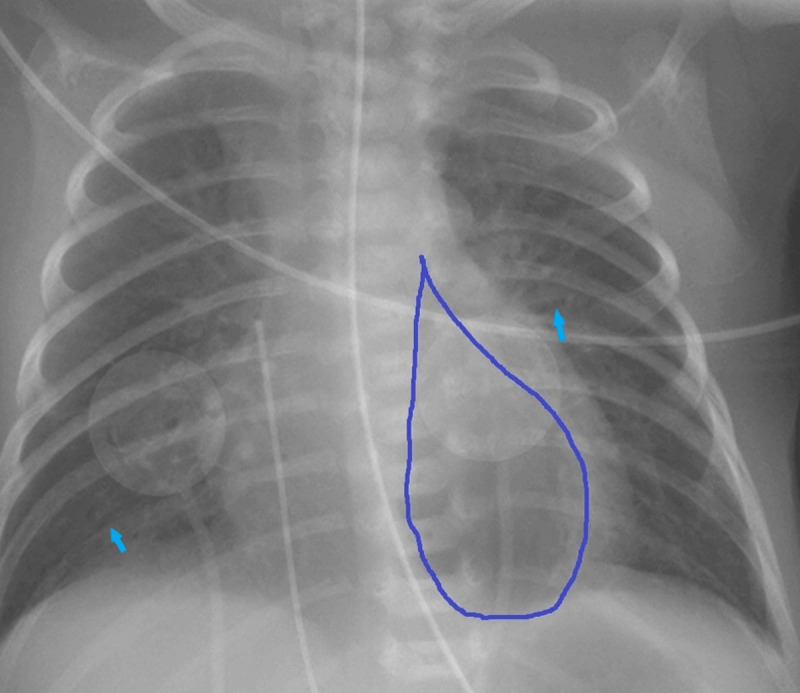


A routine frontal chest radiograph (***Figure 1***) was performed on the first day of life. It showed a waterdrop-shaped retrocardiac air lucency (***Figure 2***), which can roughly be simplified as a triangle with a summit pointing at the left pulmonary hilus and a basis lying on the left diaphragmatic dome. Its medial border was straight in a left parasagittal plane, while the lateral border was convex and bulging. This image is consistent with a trapped air collection in the pulmonary ligament.

**Figure 3:**
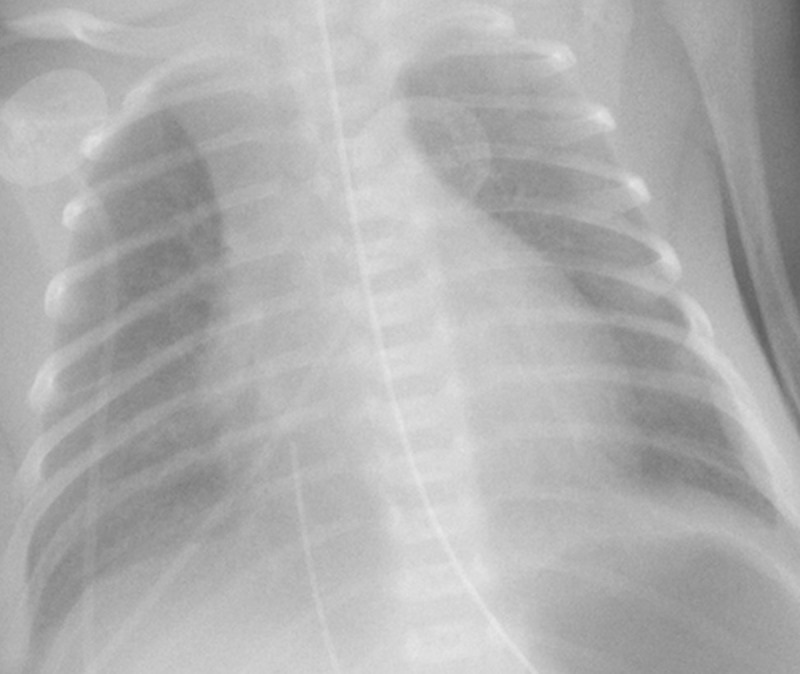


Other findings included linear radiolucent bands radiating from the hilum, consistent with pulmonary interstitial emphysema (***Figure 2***, blue arrows). There was no sign of pneumothorax. Positive pressure ventilation was diminished and a control chest radiograph performed on day 2 (***Figure 3***) demonstrated resolution of the lucency.

## COMMENT

Overdistension of immature alveoli may cause alveolar rupture with air dissection to the pulmonary interstitial space creating a pathway to the hili before eventually reaching the mediastinum and the inferior pulmonary ligaments [1].

The pulmonary ligament is a double pleural sheath, extending below the pulmonary hilus to the homolateral hemidiaphragm. It contains connective tissue that blends with the mediastinal connective tissue medially and the hilar connective tissue superiorly.

Air trapped in the pulmonary ligament in neonates often has an underlying triggering agent, usually positive pressure ventilation, associated with an underlying abnormality (e.g., surfactant deficiency) [1]. It presents as a right or left para-sagittal infrahilar oval or pyramidal lucent collection.

Some patients may develop secondary pneumothorax or even pneumoperitoneum [1], but most cases resolve spontaneously. Knowledge of this condition avoids confusion with other pathologies such as hiatal/diaphragmatic hernia or uncommon conditions such as lung pseudocysts or neonatal esophageal perforation. Timely recognition prevents the use of unnecessary diagnostic or therapeutic measures.
